# 4038 Development of a Catheter Stabilization Device for Stent Placement Aid

**DOI:** 10.1017/cts.2020.158

**Published:** 2020-07-29

**Authors:** Dylan B Crocker

**Affiliations:** 1 Duke University

## Abstract

OBJECTIVES/GOALS: Precisely, the goal of the device is to initiate a friction force between the delivery system and the arterial vessel wall to both assure immediate stent deployment and prevent axial advancement of the stent-anchoring wire. METHODS/STUDY POPULATION: A prototype was constructed and its effectiveness of applying a friction force to a vessel wall was tested ex vivo using an LRX Plus Materials Testing Machine. Afterwards, the experimental performance of the device was compared to that of a finite element simulated model. RESULTS/ANTICIPATED RESULTS: The device demonstrated the ability to apply a friction force to the vessel wall to meet its objective. However, experimental values were consistently greater than those gathered from the simulation. Since the force prescribed by the device is minimal, future work includes increasing the force capabilities of the device and defining force requirements. DISCUSSION/SIGNIFICANCE OF IMPACT: Upon further development and testing, this device can be implemented into endovascular neurosurgery to improve occlusion rates of intracranial aneurysms and reduce patient risk during these operations. CONFLICT OF INTEREST DESCRIPTION: I am pursuing intellectual property on this invention. I was careful not to describe the invention in too much detail in my abstract submission for this reason. This research is my thesis work, and I placed on one year embargo on it before it is published to give us time to sort out IP. I would like to be considered for inclusion in Translational Science 2020 if I am able to get IP on this work before publishing, which I expect will be the case. I have every intention of obtaining IP before the conference in April 2020.

